# Molecular docking analysis of imidazole quinolines with gingipain R from *Porphyromonas gingivalis*

**DOI:** 10.6026/97320630019088

**Published:** 2023-01-01

**Authors:** Kanumuru Rahul Reddy, Gayathri Rengasamy, Surya Sekaran, Vishnu Priya Veeraraghavan, Kavitha Sankaran, Rajalakshmanan Eswaramoorthy

**Affiliations:** 1 Department of Biochemistry, Saveetha Dental College and Hospitals, Saveetha Institute of Medical and Technical Science (SIMATS), Saveetha University, Chennai-600077; 2 Department of Biomaterials (Green lab), Saveetha Dental College and Hospital, Saveetha Institute of Medical and Technical Science (SIMATS), Saveetha University, Chennai-600077

**Keywords:** Antimicrobial agents, *Porphyromonas gingivalis*, imidazole quinolines, molecular docking, Insilico

## Abstract

*Porphyromonas gingivalis* is known to produce major virulence factor, Gingipain R that could penetrate the gingivae and cause tissue destruction. In this research we aim to target the gingipain R protein with imidazole quinoline derivatives (1-6) via insilico means. Molecular docking results show, compounds (1-6) have better affinity and amino acid interactions compared to the standard clinically proven drugs used as control group, and they obey Lipinski's rule of five and can be used as potential drug candidates to inhibit gingipain R.

## Background:

A Gram-negative oral anaerobe called *Porphyromonas gingivalis* plays a role in the etiology of periodontitis, an inflammatory condition that damages the tissues that support teeth and may eventually result in tooth loss. A bacterial complex known as the "red complex" made up of *Porphyromonas gingivalis*, *Treponema denticola*, and *Tannerella forsythia* has been closely linked to advanced periodontal diseases among the more than 500 bacterial species that reside in the oral cavity [1]. Numerous bacterial species that usually coexist in harmony with the host make up the microbiota of the human oral mucosa the etiological agent of severe types of periodontitis (a chronic inflammatory disease). *Porphyromonas gingivalis*, is a well-known member of the oral microbiome and an effective colonizer of the oral epithelium [2]. The initial step in the beginning of the inflammatory and immunological processes that eventually lead to the deterioration of the tissues surrounding and supporting the teeth and finally lead to tooth loss is the disruption of epithelial cells by bacteria. *Porphyromonas gingivalis* has the ability to locally penetrate periodontal tissues while dodging host defences. It accomplishes this by using a variety of virulence factors that lead to an imbalance in the innate immune system and inflammatory reactions [3, 4]. It has been shown that a wide variety of host proteins are activated and/or degraded by the cysteine proteinases (gingipains) of *Porphyromonas gingivalis*. The virulence of *P. gingivalis* is reduced when gingipains R is inactivated prior to infection of mice. The hemagglutinin domains of gingipains are highly immunogenic, according to analysis of mouse, rabbit, and chicken antisera produced to gingipain R [5]. Therefore, it is of interest to document the molecular docking analysis data of imidazole quinolines with gingipain R from *Porphyromonas gingivalis*.

## Materials and Methods:

## Protein preparation:

**Figure 1:**
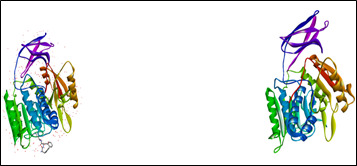
3D structure of gingipain R protein (PDB ID: 1CVR)

The 3D crystal structure of the gingipain R protein (PDB ID: 1CVR) was downloaded from the protein data bank (Figure 1). As per standard protocol, protein preparation was done using the software Biovia Discovery Studio and Mgl tools 1.5.7. Water molecules and cofactors were chosen for elimination. The previously connected ligands were removed, and the protein was produced by adding polar hydrogens and Kollmans charges with Auto Prep.

## Ligand preparations:

**Figure 2:**
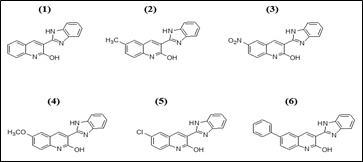
2D Structures of the imidazole quinolines compounds (1-6).

The 2D structures of the literature derived imidazole quinolines compounds are drawn using the ChemDraw 16.0 software (Figure 2). During the optimization method, the software Chem3D was employed and all parameters were selected in order to achieve a stable structure with the least amount of energy. The structural optimization approach was used to estimate the global lowest energy of the title chemical. Each molecule's 3D coordinates (PDB) were determined using optimized structure.

## Auto dock Vina analysis:

**Figure 3:**
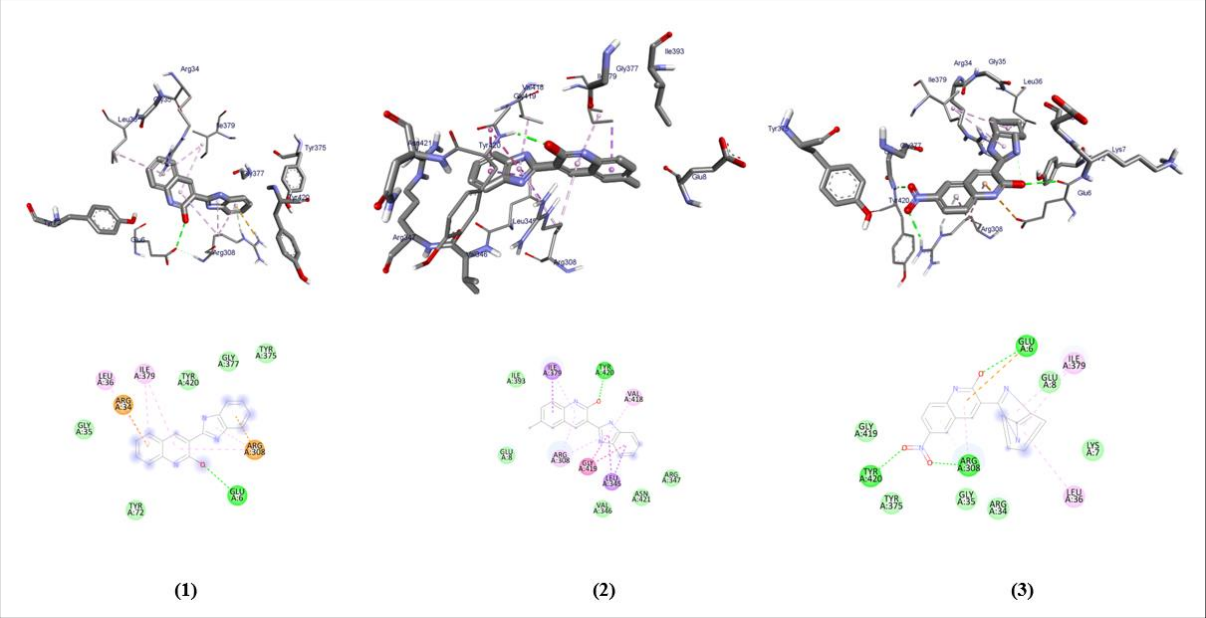
Molecular docking analysis of compounds (1-3) against the target gingipain R of *Porphyromonas gingivalis*

**Figure 4:**
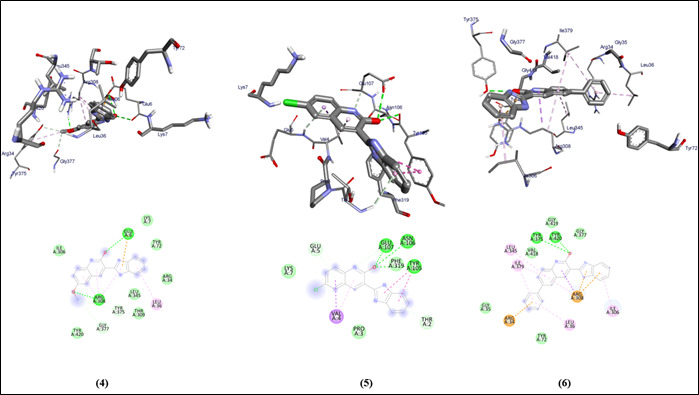
Molecular docking analysis of compounds (4-6) against the target gingipain R of *Porphyromonas gingivalis*

The graphical user interface Auto Dock vina was used for Ligand-Protein docking interactions (Figure 3,Figure 4). Auto Dock Tools (ADT), a free visual user interface (GUI) for the AutoDock Vina software, was used for the molecular docking research. The grid box was built with dimensions 54.2968, 62.9084, 42.8026 pointing in the x, y, and z axes. The central grid box for 1CVR was 63.1658, 43.1743, 57.8914 A. PyMOL utilizing Auto Dock Vina algorithms was used to analyze the interactions between the target receptor and ligands by choosing the conformations (for each ligand, nine alternative conformations were created and ranked based on their binding energies) with the most favorable (least) free binding energy [6][7][8].

## In silico Predictions of Drug-Likeness and Toxicity:

Based on the technique described by Amina et al. (2016), structures of compounds were input into the SwissADME tool and converted to their canonical simplified molecular input line entry system (SMILE) to estimate in-silico pharmacokinetic parameters and other molecular features. The total polar surface area, the amount of hydrogen donors, hydrogen acceptors, and rotatable bonds, as well as the compounds' synthetic accessibility, were all reported by the SwissADME predictor. A pharmacological agent's drug likeliness predicts and establishes if it possesses characteristics that are consistent with becoming an orally active medication. This prediction is based on the Lipinski's rule of five, a theory that Lipinski et al. have already established. The rule states that when a molecule has more than five H-bond donors, ten H-bond acceptors, a molecular weight larger than 500, and a computed LogP (CLogP) greater than five, there is likely to be poor absorption or permeation. A factor known as drug score was used to select chemicals as drug candidates. The likelihood that a compound will be regarded as a drug candidate increases with the drug score value. The ligands' and their own toxicological endpoints and organ toxicities.The probability of the compound being regarded as a drug candidate increases with the drug score value. ProTox II was used to predict the LD50 of the ligands as well as their toxicological endpoints and organ toxicities. Only substances that passed all of the screenings were used for the molecular docking study [9][10][11].

## Statistical analysis:

One way ANOVA was used for statistical analysis. The clinically proven drugs are used as a control and the results are compared. The significance of the results was found to be p<0.05 

## Results:

## Molecular docking interaction of imidazole quinolines compounds with gingipain R from *Porphyromonas gingivalis*:

All the compounds (1-6) are run against the target heme binding protein of *Tannerella forsythia* and it shows the range between -7.7 to -9.2 (Table 1). The compounds show hydrogen molecules interaction similar to clinically proven drugs used as control (Amoxicillin, Moxifloxacin, Sulfanilamide and Sulfamethoxazole. All the compounds show similar binding affinity and the lead molecules are within the binding site.

## SwissADME and Lipinski's rule of five:

The compounds show log Kp values between -4.79 to -5.88 cm/s (Table 2). All the compounds show high gastro intestinal absorption so it doesn't need a carrier molecule. Compounds show no blood brain barrier permeability. All the compounds (1-6) obey Lipinski's rule of five similar to control groups (Table 3).

## Toxicity profiling:

The compounds show class 4 toxicity. All the compounds except compounds 2 and 6 show a similar LD50 value (1000mg/kg). Compounds (1-6) are not cytotoxic (Table 4).

## Discussion:

All imidazole quinoline compounds (1-6), were chosen based on their affinity, H-bonds, and interactions with amino acids. Their structures were examined and compared with those of the common medicines. Additionally, their molecular weight, iLogP, and various other variables were checked. Gastro intestinal absorption, BBB permeability and all kinds of toxicity was checked and analyzed. Among the six compounds none was found to be cytotoxic. After considering all the parameters only three among the six compounds were further taken into consideration as the potential drug for the current study. Compounds 2, 3, and 6 were chosen among the six compounds and were compared with a standard drug moxifloxacin. Compound 6 having the highest affinity with -9.2 kcal/mol followed by compound 2 with -9 kcal/mol and then comes compound 3 with -8.4 kcal/mol. These three compounds are analyzed to be having the highest negative affinity docking score. All the three compounds have the same bio availability score which is 0.55. None of the compounds violate the Lipinski's rule. Compound 6 has the highest molecular weight and ilog P value, its molecular weight is 337.37 and ilogP value is 2.32. Compound 2 has a molecular weight of 275.3 and compound 3 has 306.28. The compound compound 2 has ilogP value of 1.82 and compound 3 has 1.3. The GI absorption is high in all the three compounds. The BBB permeability is seen in compounds 2, and 6. The PGP substrate shows positive for compounds 2, and 6 and shows negative for compound 3. Since all the compounds show inactive cytotoxicity, which is a major key point for a potential drug? All of the three compounds are hepatotoxic. Compound 3 has carcinogenicity, compounds 2, and 6 do not. Only compound 2 is immune-toxic among the three compounds and only compound 3 has mutagenicity. Thus, these compounds can be potential drugs to inhibit the factor gingipain R of *Porphyromonas gingivalis* [12, 13, 14, 15, 16, 17, 18, 19, 20].

## Conclusion:

The selected ligands show better interactions with the binding sites of the protein. All the compounds (1-6) obey Lipinski's rule of five and have excellent interaction scores when compared to already existing drugs for *Porphyromonas gingivalis*. These compounds can be taken for future studies and functional groups similar to these molecules have to be explored.

## Figures and Tables

**Table 1:** Molecular docking scores and residual amino acid interactions of Imidazole Quinoline compounds (1-6) against virulence factor gingipain R (RgpB) of *Porphyromonas gingivalis* (PDB ID – 1CVR).

Ligands	Docking scores/Affinity (kcal/mol)	H-bond	Amino Acid Residual interactions	
			Hydrophobic/Pi-Cation	Van dar Waals
1	-7.7	GLU-6	ARG-308, ARG-34, LEU-36, ILE-379	GLY-35, TYR-420, GLY-377, TYR-375, TYR-73
2	-9	TYR-420	ILE-379, VAL-418, ARG-308, GLY-419, LEU-345	ILE-393, GLU-8, VAL-346, ASN-421, ARG-347
3	-8.4	GLU-6, TYR-420, ARG-308	ILE-379, LEU-36	GLU-8, LYS-7, ARG-34, GLY-35, TYR-375, GLY-419
4	-7.7	GLU-6, ARG-308	LEU-36, TYR375, GLY-377	LYS-7, TYR-72, ARG-34, LEU-345, THR-309, TYR-420, ILE-306
5	-8	GLU-107, ASN-106, TYR-105	THR-2, GLU-5, VAL-4	LYS-7, PHE-319, PRO-3
6	-9.2	TYR-375, TYR-420	LEU-345, ILE-379, ARG-34, LEU-36, ARG-308, ILE-306	GLY-419, GLY-377, GLY-35, TYR-72
Amoxicillin	-8.1	GLY-377, GLU-6, TYR-72, ARG-34	ARG-308	THR-309, ILE-393, LEU-36, LY-35, LYS-7, GLU-8, ILE-379, GLY-419, TYR-420, LEU-345
Moxifloxacin	-8.7	ASN-376, ARG-308, TYR-420	ILE-379, LEU-36, GLY-377	
Sulfanilamide	-5.5	TYR-72, ARG-34, SER-343	ILE-379, ARG-308, LEU-345	THR-309, LEU-36
Sulfamethoxazole	-6.6	GLY-377	TYR-72, ARG-308, ARG-34, LEU-345	THR-309, GLU-6, TYR-420, GLY-419, VAL-418, ILE-379

**Table 2:** SwissADME values of selected Imidazole Quinoline compounds (1-6)

Compound	log Kp (cm/s)	GI absorption	BBB permeant	Pgp substrate	CYP1A2 inhibitor	CYP2C19 inhibitor	CYP2C9 inhibitor	CYP2D6 inhibitor	CYP3A4 inhibitor
1	-5.48	High	Yes	Yes	Yes	No	No	Yes	Yes
2	-5.31	High	Yes	Yes	Yes	Yes	No	Yes	Yes
3	-5.88	High	No	No	Yes	No	No	Yes	No
4	-5.68	High	Yes	Yes	Yes	Yes	No	Yes	Yes
5	-5.25	High	Yes	Yes	Yes	Yes	No	Yes	Yes
6	-4.79	High	Yes	Yes	Yes	Yes	No	Yes	No
Amoxicillin	-9.94	Low	No	No	No	No	No	No	No
Moxifloxacin	-8.32	High	No	Yes	No	No	No	Yes	No
Sulfanilamide	-7.79	High	No	No	No	No	No	No	No
Sulfamethoxazole	-7.21	High	No	No	No	No	No	No	No

**Table 3:** Lipinski and Veber rules of selected Imidazole Quinoline compounds (1-6)

Compound	MW	iLogP	HBD (nOHNH)	HBA (nON)	nrotb	MR	TPSA	Lipinski #violations	Bio availability score
Lipinski*	≤500	≤5	≤5	≤10	≤10	-	-		
Veber**	-	-	-	-	-	-	≤ 140		
1	261.28	1.75	2	3	1	78.85	61.8	0	0.55
2	275.3	1.82	2	3	1	83.82	61.8	0	0.55
3	306.28	1.3	2	5	2	87.68	107.62	0	0.55
4	291.3	1.86	2	4	2	85.35	71.03	0	0.55
5	295.72	1.99	2	3	1	83.86	61.8	0	0.55
6	337.37	2.32	2	3	2	104.29	61.8	0	0.55
Amoxicillin	365.4	1.46	4	6	5	94.59	158.26	0	0.55
Moxifloxacin	401.43	2.78	2	6	4	114.05	83.8	0	0.55
Sulfanilamide	172.2	0.61	2	3	1	41.84	94.56	0	0.55
Sulfamethoxazole	253.28	1.03	2	4	3	62.99	106.6	0	0.55

**Table 4:** Toxicity profile of selected Imidazole Quinoline compounds (1-6)

			Toxicity				
Compound	^a^ LD_50_ (mg/kg)	Class	HEPATOTOXICITY	CARCINOGENICITY	IMMUNOTOXICITY	MUTAGENICITY	CYTOTOXICITY
1	1000	4	Active	Inactive	Inactive	Inactive	Inactive
2	1190	4	Active	Inactive	Active	Inactive	Inactive
3	1000	4	Active	Active	Inactive	Active	Inactive
4	1000	4	Active	Active	Active	Active	Inactive
5	1000	4	Active	Inactive	Inactive	Inactive	Inactive
6	495	4	Active	Inactive	Inactive	Inactive	Inactive
Amoxicillin	15000	6	Inactive	Inactive	Inactive	Inactive	Inactive
Moxifloxacin	2000	4	Inactive	Inactive	Inactive	Active	Inactive
Sulfanilamide	3000	5	Inactive	Active	Inactive	Inactive	Inactive
Sulfamethoxazole	2300	5	Active	Active	Inactive	Inactive	Inactive
^a^ LD_50_: lethal dose parameter
